# Atrophic, translucent plaque on the scalp of a newborn

**DOI:** 10.1016/j.jdcr.2025.09.022

**Published:** 2025-10-10

**Authors:** Racquel A. Bitar, Sarah E. Servattalab, Yasin Damji

**Affiliations:** a Division of Immunology, Dermatology Section, Boston Children's Hospital, Boston, Massachusetts; b University of Massachusetts Chan Medical School, Worcester, Massachusetts; c Harvard Medical School, Boston, Massachusetts

**Keywords:** aplasia cutis, dermoscopy, infant, scalp, skin pigmentation

## Case description

**Fig 1:**
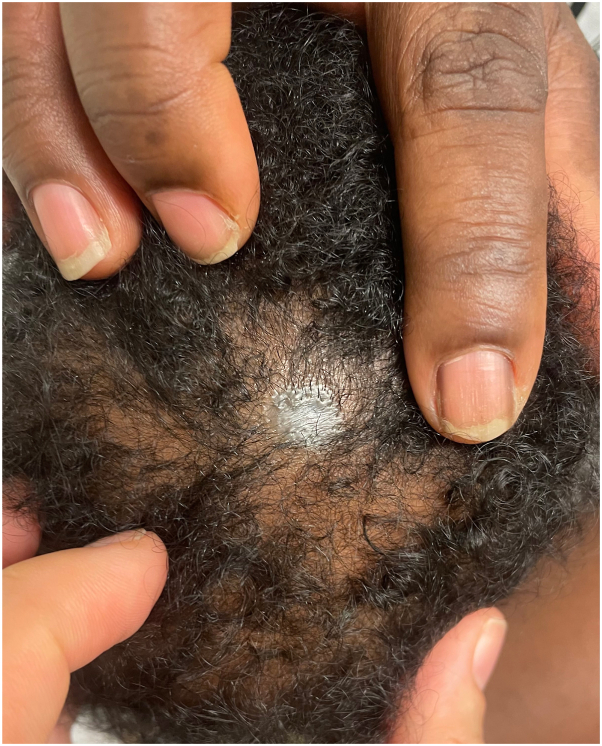
*Blue-to-gray*, atrophic, translucent plaque on the midline vertex scalp.

**Fig 2:**
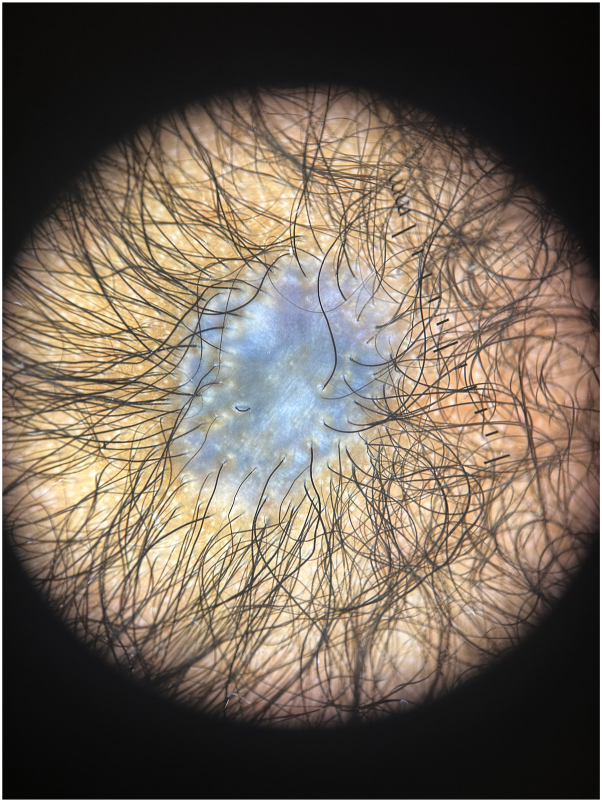
*Blue* and *gray* structureless areas and peripheral hair follicles demonstrated by dermoscopy.

A 2-month-old female was evaluated for a congenital blue plaque on the scalp. She was born at term via induced vaginal delivery for pre-eclampsia. Pregnancy was notable for gestational diabetes and intrauterine growth restriction. Medications during pregnancy included a prenatal vitamin. Growth and development were normal, and family history was unremarkable. Physical examination revealed skin phototype V and a blue-gray, alopecic, atrophic, translucent plaque without a hair collar sign on the midline vertex scalp (Fig 1). Dermoscopy revealed central blue-gray structureless areas and peripheral hair follicles (Fig 2). A head ultrasound was within normal limits and magnetic resonance imaging was not obtained.

**Question: What is the most likely diagnosis?**

**A.** Nevus sebaceus
**B.** Membranous aplasia cutis congenita
**C.** Rapidly involuting congenital hemangioma
**D.** Congenital melanocytic nevus
**E.** Epidermal nevus

**Answer: The correct diagnosis is membranous aplasia cutis congenita.**

## Discussion

Aplasia cutis congenita (ACC) is a rare dermatologic condition characterized by the absence of skin and adnexal structures.^1^ Occasionally, ACC is devoid of dermis and/or subcutis and extends to bone or fascia.^2^ Membranous aplasia cutis congenita (MACC) is the most common subtype: it presents as a discrete, ovoid, alopecic defect with an overlying shiny membrane.^1^ It is most often seen on the scalp.^1^ ACC can be isolated and sporadic, associated with other local anomalies, or part of a syndrome.^1^ The etiology of ACC remains unknown, but it has been associated with: teratogens, embryological malformations, trauma, placental infarctions, and intrauterine infection.^1^^,^^2^ Neurogenic locus notch homolog protein 3, a gene related to endothelial cell junction stabilization and neural stem cell regulation, has been implicated in 1 case of ACC.^1^ While the pathogenesis remains unknown, neurogenic locus notch homolog protein 3 can substitute for neurogenic locus notch homolog protein 1, a gene identified in Adams Oliver syndrome-related ACC.^1^

ACC is a clinical diagnosis, and skin biopsy is not usually indicated.^2^ Morphologically it can demonstrate an ulceration, scar, or membrane-covered plaque, as seen in MACC.^2^ Dermoscopy is a helpful bedside technique to make this diagnosis and rule out other conditions like trauma.^2^ Dermoscopy of MACC typically demonstrates a shiny surface with thin telangiectasias, peripheral blue globules representing hair bulbs, and the absence of hair follicles.^3^ As in this patient and others with darker skin tones, dermal pigment incontinence and dermal fibrosis may mask the aforementioned findings and instead, peripheral hair follicles can be seen.^3^ Blue-gray structureless areas were also seen on dermoscopy in this patient, which has not yet been described in MACC in skin of color. It is possible this may correlate to dermal melanocytosis originating from glial cells, which has been reported previously in ACC.^4^

Midline location and the presence of a hair collar sign, thick, rough hair surrounding the ACC, prompt further evaluation with cranial imaging to rule out underlying central nervous system or bone defects.^2^ Management of ACC primarily involves local wound care, and excision can be performed for cosmetic repair of alopecia following scar remodeling.^5^ Most superficial lesions resolve spontaneously with scar formation.^5^

This case highlights the importance of recognizing ACC in skin of color given the potential for associated anomalies. The blue-gray appearance and dermoscopic findings in this patient are distinct, and similar cases are lacking in the literature. Dermoscopy is a useful tool to confirm this diagnosis and guide further management.

## Conflicts of interest

None disclosed.
